# Antibacterial and Antifungal Activities of Poloxamer Micelles Containing Ceragenin CSA-131 on Ciliated Tissues

**DOI:** 10.3390/molecules23030596

**Published:** 2018-03-07

**Authors:** Marjan M. Hashemi, Brett S. Holden, Maddison F. Taylor, John Wilson, Jordan Coburn, Brian Hilton, Tania Nance, Shawn Gubler, Carl Genberg, Shenglou Deng, Paul B. Savage

**Affiliations:** 1 Department of Chemistry and Biochemistry, Brigham Young University, C100 BNSN, Provo, UT 84602, USA; marjan.mhashemi@gmail.com (M.M.H.); bholden222@yahoo.com (B.S.H.); maddifaytaylor@gmail.com (M.F.T.); johnmartinwilson@gmail.com (J.W.); jccoburn2@gmail.com (J.C.); hiltonb93@gmail.com (B.H.); tnance99@aol.com (T.N.); shgubler@gmail.com (S.G.); shengloudeng@hotmail.com (S.D.); 2 N8 Medical, Dublin, OH 43017, USA; carlgenberg@n8medical.com

**Keywords:** ceragenin, cilia, poloxamer, micelles, biofilm, antibacterial activity, antifungal activity

## Abstract

Ceragenins were designed as non-peptide mimics of endogenous antimicrobial peptides, and they display broad-spectrum antibacterial and antifungal activities, including the ability to eradicate established biofilms. These features of ceragenins make them attractive potential therapeutics for persistent infections in the lung, including those associated with cystic fibrosis. A characteristic of an optimal therapeutic for use in the lungs and trachea is the exertion of potent antimicrobial activities without damaging the cilia that play a critical role in these tissues. In previous work, potent antimicrobial activities of ceragenin CSA-131 have been reported; however, we found in *ex vivo* studies that this ceragenin, at concentrations necessary to eradicate established biofilms, also causes loss of cilia function. By formulating CSA-131 in poloxamer micelles, cilia damage was eliminated and antimicrobial activity was unaffected. The ability of CSA-131, formulated with a poloxamer, to reduce the populations of fungal pathogens in tracheal and lung tissue was also observed in *ex vivo* studies. These findings suggest that CSA-131, formulated in micelles, may act as a potential therapeutic for polymicrobial and biofilm-related infections in the lung and trachea.

## 1. Introduction

Ceragenins were developed as mimics of antimicrobial peptides (AMPs), and, as such, display the broad-spectrum antibacterial and antifungal activities common to most AMPs [1,2]. These activities extend to drug-resistant organisms [3,4] and to established biofilms [5,6,7]. The broad-spectrum activity of ceragenins and AMPs is due primarily to the selective association of these antimicrobials with bacterial and fungal membranes. For example, we have shown that ceragenins bind to bacterial membrane surfaces even in the presence of larger surface areas of mammalian cells [8]. This selectivity likely contributes to the antimicrobial activities of ceragenin in many tissues, without adverse effects, including the peritoneal cavity [9], in bone fractures [10], and on bone [11,12] and lung-associated [13] medical devices.

With most bacterial and fungal pathogens, ceragenins display inhibitory activity at µg/mL concentrations [1,2,3,4,6]; however, higher concentrations may be necessary to fully eradicate biofilm forms of these organisms [5,6]. In biofilm form, microorganisms enter a sessile state [14] in which some cells, including persister cells [15], remain viable even after most other cells have been eradicated by applied antimicrobials. Biofilms contribute to multiple diseases [14]. For example, infections associated with cystic fibrosis (CF) show substantial contributions from bacteria in biofilm form [16,17]. Substantial doses of antibiotics, including tobramycin, are used in an effort to suppress bacterial growth, yet biofilms persist [16,17,18].

**Figure 1:**
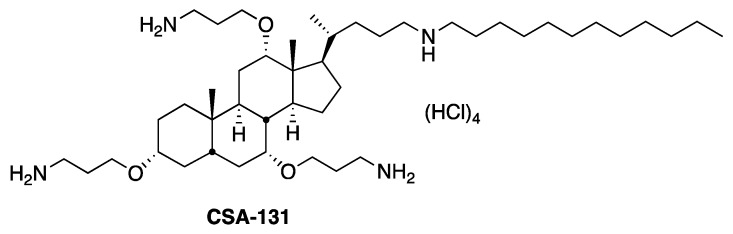
Structure of ceragenin CSA-131.

In the lungs of CF patients, bacterial biofilms accumulate along with DNA, F-actin, and mucins. These compounds deactivate innate immune defenses, including AMPs, while ceragenins remain active in their presence [19,20]. Consequently, ceragenins appear well suited for use in reducing the biofilm burden in the CF lung. The presence of endogenous AMPs in the lungs [21] suggests that tissues in the lungs are tolerant of these antimicrobials, and the fact that a ceragenin-releasing endotracheal tube is well tolerated *in vivo* [13] suggests that the lung epithelium may also be unaffected by ceragenins. However, while epithelial cells may remain viable, the cilia on these cells, which play a critical role in maintaining lung health, are fragile and can be damaged by antibiotics and mechanical insults. Consequently, evaluation of the potential use of ceragenins for the eradication of biofilms must include observation of the impact of these antimicrobials on cilia function. In initial experiments, we observed that the exposure of lungs and tracheal explants to high concentrations of a ceragenin, CSA-131 (Figure 1), led to decreases in cilia function. We therefore sought methods to decrease the impact of this ceragenin on cilia, allowing the use of concentrations of CSA-131 sufficient to eradicate biofilms without interfering with the essential role that these cilia play.

Various poloxamers have been developed for use as drug carriers. The poloxamer Pluronic^®^ F127 (hereafter referred to as pluronic) has been especially well studied due to its abilities to form well-defined micelles and sequester drugs for delivery to specific tissues [22]. In two previous studies, it was observed that combinations of this non-ionic surfactant and ceragenins decreased host cell cytotoxicity without substantially impairing antibacterial activities [23,24]. The mechanism of decreased toxicity is most likely due to transient encapsulation of ceragenins in micelles formed by the pluronic. While ensconced in micelles, ceragenins are less prone to associate with host membranes, yet the higher affinity of ceragenins for microbial membranes [8] and the dynamic nature of micelles allow ceragenins to effect antimicrobial activities. In this manner, pluronic micelles may act to amplify the membrane selectivity of ceragenins.

To observe the impact of pluronic micelles on ceragenins activity and to optimize ceragenin formulation for use in treating biofilm-based infections in the lung, we studied the impact of a lead ceragenin, CSA-131, with and without pluronic on cilia beating and the inhibition of bacterial and fungal growth *in vitro* and in tracheal and lung tissue explants. We found that the use of pluronic with relatively high concentrations of CSA-131 leaves cilia beating intact, and the pluronic has no impact on the antimicrobial activities of CSA-131. These results suggest that the formulation of ceragenin CSA-131 in pluronic micelles may allow use of high concentrations of this antimicrobial, sufficient to eliminate biofilms without negatively impacting cilia function.

## 2. Material and Methods

Ceragenin CSA-131 was prepared as previously described [25]. Pluronic^®^ F127, 2,3-bis-(2-methoxy-4-nitro-5-sulfophenyl)-2*H*-tetrazolium-5-carboxanilide and menadione were obtained from Sigma-Aldrich (St. Louis, MO, USA) and used as received.

### 2.1. Microbial Cultures

*Staphylococcus aureus* (ATCC 25923) and *Pseudomonas aeruginosa* (ATCC 47085 (PA01)) were grown from fresh colonies in trypticase soy broth (TSB) and incubated overnight at 37 °C. Fungal cultures, *Candida albicans* (ATCC 90028) and *Candida auris* (CDC 0382, 0384, 0387, and 0389), were grown overnight in sabouraud dextrose broth (SDB) or Roswell Park Memorial Institute medium (RPMI). Cultures of bacteria and fungi were centrifuged, and pellets were washed three times with phosphate buffered saline (PBS) and further resuspended in fresh PBS. Bacterial cultures were diluted in TSB and fungal cultures were diluted in SDB or RPMI to 10^3^ or 10^6^ CFU/mL (optical density (OD) readings at 600 nm).

### 2.2. Susceptibility Testing in the Presence or Absence of Pluronic

#### 2.2.1. Minimum Inhibitory Concentration (MIC)

MICs were measured using the broth microdilution protocol described by the Clinical and Laboratory Standards Institute [26,27]. Briefly, two-fold dilutions of CSA-131 were dispensed in separate wells of a 96-well plate. Aliquots (100 µL) of a prepared inoculum (10^6^ CFU/mL for bacteria and 10^3^ CFU/mL for fungi) were added, and plates were incubated at 37 °C for 18–20 h. Bacterial or fungal growth was visually observed to determine the MICs. Negative and positive controls were included for each set of MIC measurements. For studies with pluronic, the surfactant was added at 4% and 5% to the initial inocula in each experiment. Measurements were performed in triplicate for all susceptibility tests.

#### 2.2.2. Determination of Antifungal and Antibacterial Susceptibilities of Biofilms by XTT Assay

Biofilm formation by selected bacterial and fungal strains was quantified by measuring metabolic activity within biofilms using 2,3-bis-(2-methoxy-4-nitro-5-sulfophenyl)-2*H*-tetrazolium-5-carboxanilide (XTT) [28]. Biofilms were formed in 96-well plates and incubated for 48 h at 37 °C. After three washes with PBS to remove planktonic cells, CSA-131 (100 µg/mL) in the presence or absence of 4% or 5% pluronic was added to the wells and incubated for 24 h. After incubation, the wells were carefully washed with PBS. A solution of 10 mM menadione in 100% acetone was added to an XTT solution (0.5 mg/mL) and mixed. An aliquot of the XTT/menadione solution (100 µL) was added to each well. Each plate was then wrapped in aluminum foil and incubated for 2–3 h at 37 °C. To prepare to read the optical density, aliquots of the supernatant (70 µL) were removed from each well. Using a microtiter plate reader, colorimetric changes were measured at 490 nm. The percent of biofilm survival for each well containing CSA-131 with or without pluronic was calculated in comparison to the biofilm formed in the absence of the ceragenin (control).

#### 2.2.3. Determination of Minimum Biofilm Eradication Concentrations (MBEC)

Inocula (10^6^ CFU/mL) of bacterial and fungal strains (*P*. *aeruginosa*, *S*. *aureus*, *C. albicans*, and *C. auris*) were incubated at 37 °C for 48 h in 1 mL of TSB or SDB in 96-well plates. Following incubation, the growth medium was gently removed, and wells were washed three times with sterile PBS. CSA-131 (100 µg/mL), in TSB or SDB and in the presence or absence of pluronic (4% or 5%), was added to the wells at concentrations ranging from 1 to 256 µg/mL, and each plate was incubated at 37 °C for 24 h. Wells, including well edges, were scraped thoroughly with a plastic spatula. Well contents were removed in 1 mL of neutralizing broth (Dey-Engley, Sigma–Aldrich) and placed in a sonicating water bath (Fisher Scientific FS60, 42 kHz, 100 W, Pittsburg, PA, USA) to disrupt biofilms. After 15 min, the resulting samples were serially diluted, and bacterial samples were plated on TSA while fungal samples were plated on SDA. After 24 h or 48 h of incubation at 37 °C, colonies were counted and MBECs were determined. The MBEC was defined as the lowest concentration of antibiotic that prevented bacterial regrowth [29].

#### 2.2.4. Measurement of Kinetics of Bactericidal and Fungicidal Activity

Cultures of *S*. *aureus*, *P*. *aeruginosa*, and *C. albicans* were prepared as described for MIC measurements and placed in 96-well plates (10^6^ CFU for bacteria and 10^3^ CFU for fungi) with or without CSA-131 (100 µg/mL), with or without pluronic (4%), in TSB (with bacteria) or SDB (with fungi). Cultures were incubated at 37 °C. At 5, 15, 30, 60, and 120 min, aliquots were removed from each well and added to neutralizing buffer to ensure that no further antibacterial activity occurred. These samples were then serially diluted and plated on TSA or SDA. Cultures were incubated at 37 °C for 24 h or 48 h, and colonies were counted and recorded.

### 2.3. Preparation of Tracheal Explants

Normal healthy porcine trachea and lung tissue was excised very fresh from slaughter. After aseptic collection, whole tissues were transported in a 1:1 mixture of Dulbecco’s modified Eagle’s medium (DMEM) and RPMI, which was pre-warmed and supplemented with penicillin-streptomycin-glutamine 100X (Hyclone, Logan, UT, USA). Explants were washed by tissue immersion three to four times in warm, fresh DMEM/RPMI media. Explants were maintained by continuous immersion in DMEM/RPMI media in a 5% CO_2_–95% air mixture in a humidified incubator at 37 °C. Ethics statement: Trachea and lung materials were obtained from a local butcher (Circle V Meat Co., Spanish Fork, UT, USA) and were sourced from animals slaughtered for human consumption; hence, ethical approval was not required for this research.

### 2.4. Latex Bead Clearance Assay

Tracheas were washed and extra exterior tissues to the cartilage were removed. Tracheas were opened and cut into approximately 2 × 1 cm explants consisting of the respiratory mucosa and underlying cartilage. Explants were then immersed in different concentrations of CSA-131, with and without pluronic, in the DMEM/RPMI mixture in 24-well plates for 1 h in a humidified incubator at 37 °C with 5% CO_2_. Explants were then placed on small circular filter papers, which were placed on top of 1% agarose gel plugs bathed in medium in 6-well plates. To determine cilia activity on explants, epithelial surfaces were tested via a bead clearance assay 1 h post treatment with CSA-131 (100 µg/mL), with or without pluronic (1%, 2%, 3%, 4%, and 5%). Five microliters of a suspension of 1 µm diameter polystyrene microsphere beads (Polysciences, Inc., Warrington, PA, USA) were pipetted on one edge of epithelial surfaces and evaluated for their complete clearance (movement) across the surface with categorization of clearance in under 10 min, between 10 and 20 min, between 20 and 30 min, and no clearance (>30 min). Independent replicates were completed for each time point. Outcomes were then counted and a percentage of clearance was determined [30].

### 2.5. Scanning Electron Microscopy of Cilia on Porcine Trachea

Explants (5 mm^3^) were washed gently in Sorensen buffer (0.1 M, pH 7.2) to remove mucus and secretions. The fixative compounds were all diluted in the Sorensen buffer. The trachea pieces were incubated for 24 h with 2% of glutaraldehyde (Electron Microscopy Sciences, Hatfield, PA, USA) at 4 °C and then rinsed five times in Sorensen buffer. Additional post-fixation was performed using 1% of OsO_4_ (Electron Microscopy Sciences, Hatfield, PA, USA) for 1.5 h under the hood at room temperature. After fixation, OsO_4_ was removed by rinsing the explants at least seven times in Sorensen buffer and then explants were dehydrated in increasing concentrations of ethanol: 10, 30, and 50, each step for 15–20 min. The process was completed inside critical dryer baskets with 70% and 95% ethanol finishing twice in 100% for 15–20 min. Samples were then submerged in 100% ethanol in the critical point dryer at the critical point of carbon dioxide. Prepared samples were mounted on aluminum stands and then sputter-coated with 5–10 nm of a gold-palladium alloy and observed with SEM (FEI Helios NanoLab 600 SEM/FIB, Hillsboro, OR, USA) at 5 kV.

### 2.6. Ex Vivo Efficacy Evaluation

Using 5 mm^3^ explant cubes of porcine trachea and lung, *ex vivo* antifungal efficacy experiments were conducted. Cubes were dissected using a sterile razor blade form the ventral surface of the left caudal lobe of three sets of porcine lungs [31]. After trimming excess tissues away from the explants to produce uniform of size (5 mm), explants were incubated at 37 °C in DMEM/RPMI inoculated with 10^6^ CFU/explant of *C. auris* (CDC 0384) or *C. albicans* (ATCC 90028) in a 48-well plate. Explants were treated after 2 h of incubation with CSA-131 in the presence or absence of pluronic. Treated explants were incubated for 24 h at 37 °C. After incubation, tissues were suspended in 250 µL of neutralizing broth and vortex on the highest setting for 4 min. Samples were then serially diluted in PBS, plated on 5% sheep blood TSA plates, and incubated at 37 °C for 48 h [32].

## 3. Results and Discussion

We initially determined the impact of pluronic, at 4% and 5% of the growth medium, on the antibacterial and antifungal activity of ceragenin CSA-131. The amphiphilic nature of the ceragenin makes it likely that it associates well with pluronic micelles. At issue is whether the ceragenin can effectively escape micelles to exert antimicrobial effects. Earlier reports with bacteria [23,24] demonstrated that pluronic did not substantially affect antibacterial activity. The MICs and MBECs of CSA-131 alone and in the presence of pluronic at 4% and 5% are given in Table 1. MBECs are the concentrations required to eliminate biofilms, including persister cells, to detection limits. With both *S*. *aureus* and *P*. *aeruginosa*, MICs were unchanged in the presence of pluronic, as observed previously. Notably, *Candida* spp. MICs were also unaffected by pluronic, even though these are eukaryotic organisms. Notably, MBECs with both bacteria and fungi were unchanged in the presence of pluronic.

**Figure 2:**
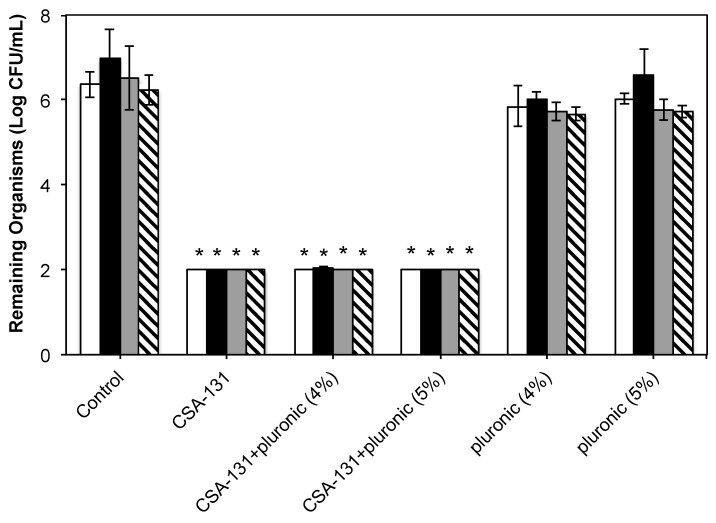
Antibiofilm results determined through the plating of microorganisms freed from biofilms, culturing, and plate counting. White bars: *S. aureus* (ATCC 25923); black bars: *P. aeruginosa* (ATCC 47085); gray bars: *C. albicans* (ATCC 90028); hashed bars: *C. auris* (CDC 384). Detection limit: 2 logs. * indicates *p* < 0.05 relative to controls and to pluronic alone.

Results from studies given in Table 1 and a previous study by Nagant et al. [5,23] suggest that a concentration of ceragenin of 100 µg/mL is sufficient to eradicate established bacterial and fungal biofilms. Consequently, we used this concentration as a target for antibacterial and antifungal activity, as well as in tolerability studies with trachea and lung explants. A key preliminary question in these studies is whether the pluronic alone has any effect on bacteria and fungi. To answer this question, the target concentration of CSA-131 (100 µg/mL) was prepared with and without pluronic, and antibiofilm activity was measured (Figure 2). In addition, established biofilms were exposed to pluronic alone. In these assays, biofilms were generated over 48 h, and after treatment, bacterial and fungal counts were determined by plating organisms freed from biofilms and counting colonies. Detection limits for the experiments were two logs. At 100 µg/mL, with and without pluronic, CSA-131 lowered bacterial and fungal counts to the detection limit (>four-log reduction (99.99% reduction)). Pluronic alone did not significantly influence microbial counts from the biofilms.

**Figure 3:**
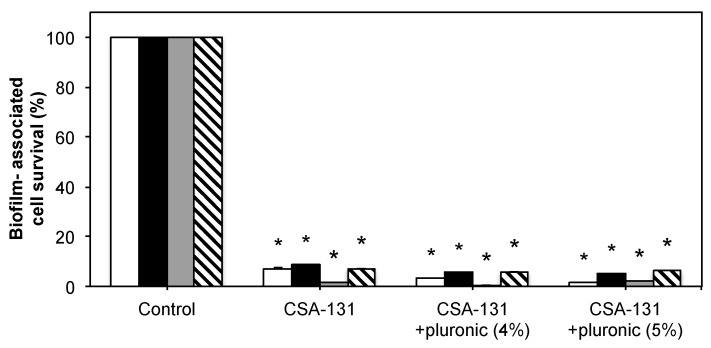
Antibiofilm results determined through colorimetric (XTT) assay represented as percent survival. White bars: *S. aureus* (ATCC 25923); black bars: *P. aeruginosa* (ATCC 47085); gray bars: *C. albicans* (ATCC 90028); hashed bars: *C. auris* (CDC 384). * indicates *p* < 0.05 relative to controls.

To corroborate the antibiofilm data from counting bacterial and fungal colonies, a colorimetric assay was performed to quantify biofilms remaining on surfaces after treatment. Results were normalized to untreated controls (Figure 3). Significant decreases in biofilm were observed, but differences between CSA-131 alone and with pluronic were not significant.

**Figure 4:**
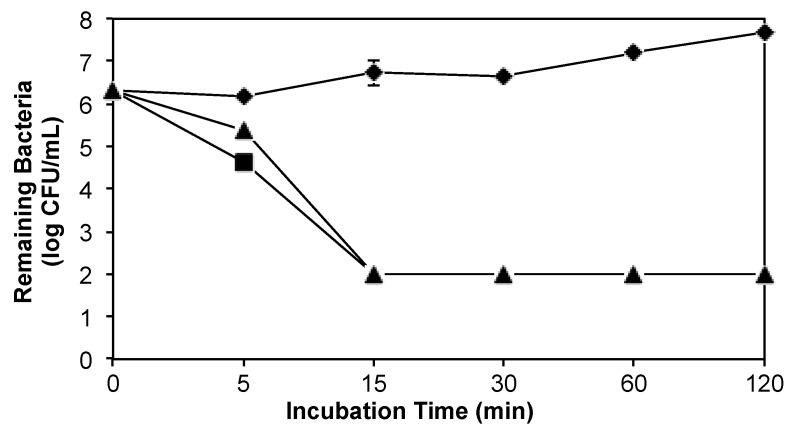
Kinetic antibacterial activity against *P. aeruginosa* of CSA-131 (100 µg/mL) (black squares), CSA-131 (100 µg/mL) with pluronic (4%) (black triangles); untreated control (black diamonds). Detection limit was two logs.

A possible consequence of the association of CSA-131 with pluronic micelles would be the slowing of the kinetic antimicrobial activities of this ceragenin relative to the ceragenin alone. We measured bacterial and fungal counts over time with CSA-131 (100 µg/mL) and CSA-131 with pluronic (4%). Results from an experiment with *P. aeruginosa* (ATCC 47085) are shown in Figure 4. Inocula of just over 10^6^ CFU/mL were reduced to the detection limit within 15 min. At 5 min, there was a small difference in bacterial counts with CSA-131 alone and CSA-131 with pluronic. With *S. aureus* and with *C. albicans*, no significant differences between kinetic activity of the ceragenin alone and with pluronic were observed (data not shown).

**Figure 5:**
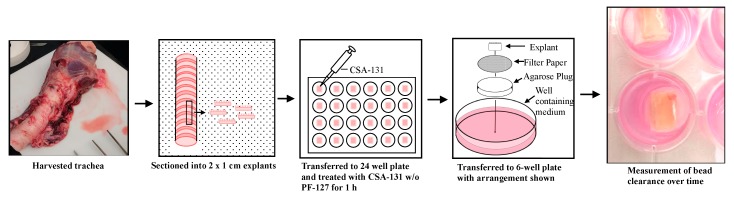
Description of methods used in harvesting and testing porcine trachea explants.

Having established the antibiofilm activity of CSA-131 in the presence of pluronic, we determined the impact of formulations of CSA-131 on ciliated tissue explants from porcine tracheas. The cilia on these tissues play a critical role in removing particulate matter from the lung and trachea; consequently, it was important to establish that CSA-131 can exert antibacterial and antifungal activity without damaging cilia. Furthermore, the presence of undamaged cilia is an indication that the underlying epithelial and goblet cells are unaffected by ceragenin treatment. An *ex vivo* assay was used to evaluate pluronic-containing formulations, and in this assay the beating of cilia can be actively observed. These observations were used to determine the concentration of pluronic, with CSA-131 (100 µg/mL), that would not impact cilia function, and by extension leave undamaged the underlying epithelial and goblet cells [31]. This method involves sectioning porcine trachea, supported in a nutrient medium, placing small beads on one side of the explant, and measuring the transfer (clearance) of the beads to the other side of the explants (Figure 5). In our hands, we could reproducibly measure bead clearance one hour after sectioning and treatment. Twelve explants were used for each test condition, and the results are reported as the number clearing beads in a given amount of time.

With no treatment with CSA-131, all 12 explants cleared the applied beads within 20 min, and 10 of the explants cleared the beads in less than 10 min (Table 2). Treatment of the explants with CSA-131 at 100 µg/mL for one hour caused a majority (six of 12) to lose the ability to clear the beads. Increasing amounts of pluronic (from 1 to 5%) restored the ability of the cilia to clear the beads, and at 5% pluronic, bead clearance was the same as the control.

**Figure 6:**
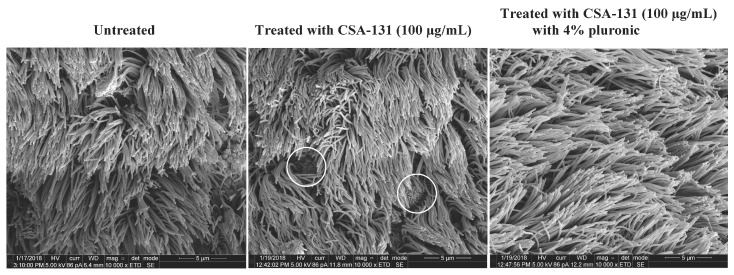
SEM images of cilia on porcine trachea explants untreated, treated with CSA-131 at 100 µg/mL, and treated with CSA-131 at 100 µg/mL with 4% pluronic. Exposed goblet cells are circled in the image of the sample treated with CSA-131 without pluronic.

To visualize the impacts of treatment of CSA-131, with and without pluronic, on cilia, we obtained scanning electron microscope (SEM) images of explants that had been treated with CSA-131 (100 µg/mL) alone and with pluronic (4%) (Figure 6). With the untreated explant, intact cilia were observed without exposure of the underlying goblet or epithelial cells. In the image of the explant treated with CSA-131 alone, exposed goblet cells were observed, suggesting that some loss of cilia had occurred. This loss correlated with decreased cilia function in the bead clearance assay. In contrast, with the explant treated with CSA-131 with pluronic (4%), a fully intact cilia bed was observed with no exposure of goblet or epithelial cells. This treatment did not influence bead clearance, and also it did not appear to influence the cilia bed. Dias et al. observed a similar loss of cilia and concomitant exposure of goblet cells in canine trachea from intubated dogs [33].

**Figure 7:**
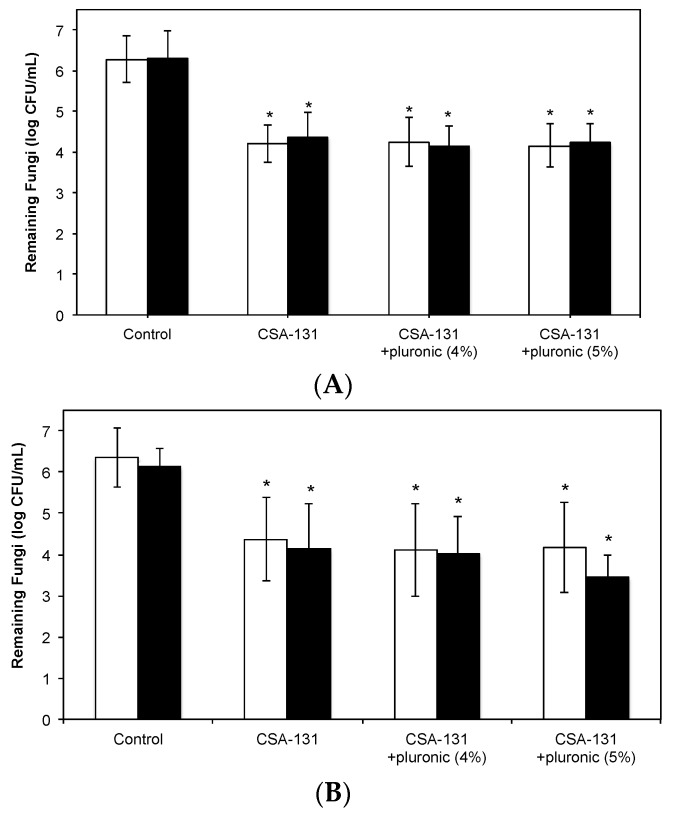
Fungi remaining in tissue explants after incubation for two hours followed by treatment with CSA-131 (with and without pluronic (4% or 5%) for one hour. White bars: *C. albicans* (ATCC 90028); black bars: *C. auris* (CDC 384). (**A**) trachea explants; (**B**) lung explants.

We next verified that CSA-131, formulated with pluronic, was able to eradicate microorganisms in tissue from the trachea and lung. For these studies, explants were infected with either *C. albicans* or *C. auris* then treated with CSA-131 alone or with pluronic (4% or 5%). In control explants, fungal counts were over six logs in both tissue types (Figure 7). From treated explants, fungal counts were one and a half to two logs less (a decrease of up to 99%). Differences from controls were statistically significant (*p* < 0.05), but small differences between results with CSA-131 alone and CSA-131 with pluronic (4% or 5%) were not significant. Interestingly, there was no significant difference of fungal growth in the tracheal and lung explants.

## 4. Conclusions

Bacteria and fungi are both important pathogens in the trachea and lung, and infections are increasingly recognized as polymicrobial. Consequently, effective development of new therapies should target a broad spectrum of bacteria and fungi. In addition, persistent infections, including those associated with CF, involve biofilm components, which adds an antibiofilm component to therapy development. AMPs play important roles in controlling microbial growth in these tissues; however, it is evident that in some situations these innate immune defenses fail to fully protect the trachea and lung. Considerations of means of augmenting the activities of AMPs are complicated by the susceptibility of cilia to damage, and since they play vital roles in these tissues, potential treatments must take into account impacts on cilia. Ceragenins have been shown to have potent activity against pathogens associated with the trachea and lung, including the ability to eradicate established biofilms. Nevertheless, concentrations of ceragenin CSA-131 necessary to eliminate biofilms cause cilia damage. To mitigate these effects on cilia, we employed the poloxamer Pluronic^®^ F-127. The association of CSA-131 with micelles formed by this surfactant does not alter antimicrobial activity against planktonic or biofilm forms of targeted microorganisms. However, with pluronic, the impact of CSA-131 on cilia is abolished. *Ex vivo* studies of CSA-131 with pluronic demonstrate the ability of this ceragenin to substantially reduce numbers of pathogenic strains of fungi in tissue. As a mimic of AMPs and with the protective effects of pluronic, ceragenin CSA-131 appears to be well suited for the treatment of polymicrobial and biofilm-related infections.

## Figures and Tables

**Table 1:** Minimum inhibitory concentration (MIC) and minimum biofilm eradication concentration (MBEC) of CSA-131 with bacteria and fungi in the absence or presence of pluronic.

MIC (MBEC) (µg/mL)			
Strains	CSA-131	CSA-131 + 4% Pluronic	CSA-131 + 5% Pluronic
***S. aureus*** **ATCC 25923**	1 (100)	1 (100)	1 (100)
***P. aeruginosa*** **ATCC 47085**	2 (100)	2 (100)	2 (100)
***C. auris*** **CDC 382**	0.5 (82)	0.5 (82)	0.5 (82)
***C. auris*** **CDC 384**	0.5 (48)	0.5 (48)	0.5 (48)
***C. auris*** **CDC 387**	0.5 (64)	0.5 (64)	0.5 (48)
***C. auris*** **CDC 389**	0.5 (48)	0.5 (48)	0.5 (48)
***C. albicans*** **ATCC 90028**	0.5 (48)	0.5 (48)	0.5 (48)

**Table 2:** Number of explants (out of 12) clearing beads during the indicated amount of time after incubation with CSA-131 (100 µg/mL) for 1 h. The control was not treated with CSA-131. Indicated amounts of pluronic were used with ceragenin.

Time Required for Bead Clearance				
Pluronic	<10 min	<20 min	<30 min	No Clearance
Control	10/12	2/12	-	-
0%	3/12	2/12	1/12	6/12
1%	4/12	2/12	1/12	5/12
2%	7/12	3/12	2/12	-
3%	7/12	4/12	1/12	-
4%	8/12	4/12	-	-
5%	10/12	2/12	-	-
